# Right temporal cortical hypertrophy in resilience to trauma: an MRI study

**DOI:** 10.3402/ejpt.v7.31314

**Published:** 2016-07-28

**Authors:** André Sevenius Nilsen, Eva Hilland, Norunn Kogstad, Trond Heir, Edvard Hauff, Lars Lien, Tor Endestad

**Affiliations:** 1 Institute of Psychology, University of Oslo, Oslo, Norway; 2 Diakonhjemmet Hospital, Oslo, Norway; 3 Innlandet Hospital Trust, Brumunddal, Norway; 4 Institute of Clinical Medicine, University of Oslo, Oslo, Norway; 5 Norwegian Center for Violence and Traumatic Stress Studies, Oslo, Norway; 6 Division of Mental Health and Addiction, Oslo University Hospital, Oslo, Norway; 7 Faculty of Public Health, Hedmark University College, Elverum, Norway

**Keywords:** Psychotraumatology, PTSD, natural disaster, traumatic event, psychological distress, MRI

## Abstract

**Background:**

In studies employing physiological measures such as magnetic resonance imaging (MRI), it is often hard to distinguish what constitutes risk-resilience factors to posttraumatic stress disorder (PTSD) following trauma exposure and what the effects of trauma exposure and PTSD are.

**Objective:**

We aimed to investigate whether there were observable morphological differences in cortical and sub-cortical regions of the brain, 7–8 years after a single potentially traumatic event.

**Methods:**

Twenty-four participants, who all directly experienced the 2004 Indian Ocean Tsunami, and 25 controls, underwent structural MRI using a 3T scanner. We generated cortical thickness maps and parcellated sub-cortical volumes for analysis.

**Results:**

We observed greater cortical thickness for the trauma-exposed participants relative to controls, in a right lateralized temporal lobe region including anterior fusiform gyrus, and superior, middle, and inferior temporal gyrus.

**Conclusions:**

We observed greater thickness in the right temporal lobe which might indicate that the region could be implicated in resilience to the long-term effects of a traumatic event. We hypothesize this is due to altered emotional semantic memory processing. However, several methodological and confounding issues warrant caution in interpretation of the results.

**Highlights of the article:**

- Following a traumatic event, most people do not develop long-lasting trauma-related symptoms.
- In a group who experienced a traumatic event 8 years prior, but showed low levels of trauma-related symptoms, we observed increased cortical thickness in the right temporal lobe.
- The right temporal lobe is implicated in emotional semantic memory processing, and thus might be associated with resilience to the long-term effects of a traumatic event.

Posttraumatic stress disorder (PTSD) is caused by major life stressors such as accidents, war, or perceived threat to life. However, most people do not develop PTSD after a potential traumatic event (Kessler, 1995). Several factors predict susceptibility to PTSD such as age, gender, socio-economic status (Perkonigg, Kessler, Storz, & Wittchen, 2000), other life stressors, and comorbid mental disorders such as depression (Brewin, Andrews, & Valentine, 2000). Prevalence rates also vary between different kinds of event types, occupational roles of survivors, and even geographic locations (Berger et al., 2012; Darves-Bornoz et al., 2008; Norris, 1992). Most of these factors fall along the risk-resilience spectrum, as they are unlikely to change due to trauma exposure; however, when investigating physiological measures such as brain morphology, it is more difficult to distinguish what constitute risk-resilience factors and what the effects of trauma exposure and PTSD are.

Morphological studies of individuals with PTSD have implicated several regions, mostly along the fear circuitry model, including amygdala, hippocampus, prefrontal cortex (PFC), and anterior cingulate cortex (ACC) (Kühn & Gallinat, 2013; Rauch, Shin, & Phelps, 2006; Shin & Handwerger, 2009). Several PTSD studies have also focused on the association between degree of trauma and morphology. One recent magnetic resonance imaging (MRI) study investigated people at various distances from 9/11 and observed an association between symptom load and gray matter volume in the amygdala, hippocampus, insula, PFC, and ACC, dependent on how far away subjects were from the twin towers (Ganzel, Kim, Glover, & Temple, 2008). A study of survivors in a coal mine accident found a negative association between the clinician-administered PTSD scale (CAPS) scores in PTSD-positive subjects and gray matter volume in the ACC (Chen et al., 2012). Gray matter volume of the frontal and temporal lobes has been implicated in combat veterans with PTSD (Geuze, Westenberg, et al., 2008), and the researchers observed a dissociation between cortical thickness and memory performance in PTSD-positive participants, but an association for PTSD-negative participants. In addition to these studies, a meta-analysis including nine studies of diverse forms of trauma found that PTSD-positive vs. PTSD-negative participants had lower gray matter volume in ACC, venteromedial PFC, left temporal pole/middle temporal gyrus, and left hippocampus (Kühn & Gallinat, 2013). On the contrary, Landré et al. (2010) observed no differences between non-combat PTSD-positive and healthy controls which cautions against assuming that results from one kind of trauma automatically generalizes to the next. In summary, most studies observe morphological differences in the hippocampus, amygdala, ACC, and PFC. However, similar regions have also been observed in studies of trauma survivors without PTSD.

Comparisons between trauma-exposed (TE) individuals without PTSD and healthy controls have revealed several regions of interest; a meta-analysis by Karl et al. (2006) implicated the amygdala, hippocampus, ACC, corpus callosum, and the frontal lobe, whereas another meta-analysis by Smith (2005) implicated hippocampus, while a recent review by O'Doherty et al. (2015) implicated the hippocampus and ACC, but not the amygdala. In summary, the amygdala, hippocampus, ACC, and prefrontal regions are heavily implicated in both the PTSD-positive and the PTSD-negative literature. Assuming that trauma exposure alone is not enough to cause observable longitudinal group differences in morphology, as indicated by a seminal prospective study by Van Wingen, Geuze, Vermetten, and Fernández (2011), one hypothesis is that several of the above-implicated areas might be associated with risk-resilience factors rather than effects of trauma.

For instance, the PFC has been implicated as a resilience factor to stress and anxiety in rodents (Russo, Murrough, Han, Charney, & Nestler, 2012), and in another study on soldiers resilient to combat-related PTSD, researchers observed decreased activity in PFC and nucleus accumbens, and abnormal plasticity levels in hippocampus, amygdala, and PFC (Wu et al., 2013). A recent review on risk factors for PTSD also implicated hippocampus, PFC, ACC, posterior cingulate cortex, temporal gyrus, and amygdala, as risk factors to PTSD development and severity (Schmidt et al., 2015).

However, most studies are retrospective with regard to the traumatic event, and thus, it is difficult to distinguish between causal effects and risk-resilience factors, although some studies have approached the problem differently. For instance, a study by Gilbertson et al. (2002) compared monozygotic twins where one had served in the Vietnam War and the other had not. The results showed that smaller hippocampus predicted PTSD symptom load; however, this was also the case for the non-combat exposed twins, suggesting that smaller hippocampal volume constitute a risk factor rather than as a PTSD-specific effect (Gilbertson et al., 2002). In addition, hippocampus size has also been observed as a factor in remission from PTSD (Van Rooij et al., 2015), indicating hippocampus size as a resilience factor to persistent PTSD, and might also be a factor in resilience to PTSD.

To investigate risk-resilience factors and causal effects, one should ideally employ longitudinal prospective trauma studies or monozygotic twin studies; however, such studies are challenging. On the contrary, investigating trauma survivors several years after a traumatic event with high levels of reported PTSD prevalence, and focusing on individuals with low current symptom load, might provide insight into resilience and remission factors, as such a sample would constitute remitters and unaffected resilient individuals. In the present project, we aimed to explore long-term cortical and sub-cortical morphological changes in participants who have previously experienced a potentially traumatic event, compared with healthy controls. In addition, we aimed at focusing on individuals with current light symptom load.

## Methods

### Participants

Twenty-five participants from an interview study of 63, out of 82 (Hussain, Weisaeth, & Heir, 2011), accepted to participate in the study. Participants were all Norwegian disaster survivors who were in Khao Lak, Thailand, at the time of the 2004 South Asian tsunami. PTSD prevalence in the sampled population was reported at 36.5%, 30 months after the disaster (Hussain et al., 2011).

Inclusion criteria for the TE group: directly experienced the 2004 Tsunami and were above 18 years at the time of the disaster. Exclusion criteria for both groups: history of head trauma, MRI incompatible implants or conditions, cerebral infection, dyslexia, serious medical or neurological illness, non-functional Norwegian skills, and organic mental and psychotic disorders.

One participant was under 18 years at the time of the disaster and was excluded from the study, leaving 24 TE (male=14, age=48.4, SD=11.1) who directly experienced the 2004 South East Asia Ocean Tsunami 7–8 years prior. Twenty-five healthy controls (HC; male=12, age=46.9, SD=21.2) were recruited from written adverts. Participants signed informed consent forms, underwent scanning, and then answered three questionnaires. All participants were compensated for travel costs and lost work hours.

### Stimuli

Participants completed Becks Depression Inventory (BDI; Beck, Steer, & Carbin, 1988), Becks Anxiety Inventory (BAI; Beck, Epstein, Brown, & Steer, 1988), and Impact of Event Scale—Revised (IES-R; Weiss & Marmar, 1997). Data were aggregated according to the specific questionnaires’ coding-schemes and between group differences were analyzed in the Statistical Package for the Social Sciences (SPSS; v22).

### Setup

The scans were performed at Ullevål University Hospital (Oslo, Norway) on a 3 Tesla Signa HDxT Siemens Scanner, using an 8-channel head coil. The imaging protocol consisted of a three-dimensional (3-D) T1-weighted sequence (Magnetization Prepared Rapid Acquisition Gradient Echo; MPRAGE) covering the entire head and having the following image parameters: voxel dimensions 1×1×1 mm and 0.2 mm slice gap, reconstructed into a 256×256×166 matrix, 2.9 s echo time, 7.7 s repetition time, and 12° flip angle.

### Structural analysis

Cortical reconstruction and volumetric segmentation was performed with the Freesurfer image analysis suite (v5.3; www.surfer.nmr.mgh.harvard.edu). Briefly, this processing includes motion correction and averaging (Reuter, Rosas, & Fischl, 2010) of volumetric T1-weighted images, removal of non-brain tissue using a hybrid watershed/surface deformation procedure (Ségonne et al., 2004), automated Talairach transformation, segmentation of the subcortical white matter and deep gray matter volumetric structures (Fischl et al., 2002), intensity normalization (Sled, Zijdenbos, & Evans, 1998), tessellation of the gray matter and white matter boundary, automated topology correction (Fischl, Liu, & Dale 2001), and surface deformation following intensity gradients to locate the gray/white and gray/cerebrospinal fluid borders (Fischl & Dale, 2000). After the cortical models, the surface was inflated (Fischl, Sereno, Tootell, & Dale, 1999), registered to a spherical atlas which utilized individual cortical folding patterns to match cortical geometry across subjects (Fischl, Sereno, & Dale, 1999), parcellation of the cerebral cortex into units based on gyral and sulcal structure (Desikan et al., 2006), and creation of a variety of surface-based data including maps of curvature and sulcal depth.

The individual cortical thickness results were imported in Freesurfer's Qdec GUI and analyzed with 10 mm FWHM smoothing using a general linear model (GLM). Age being a significant factor in cortical thickness and sub-cortical volume (Lemaitre et al., 2012), it was included as a covariate in the model. Cortical clusters were thresholded at an a-priori uncorrected significance threshold of *P*<0.001. To avoid inflation of type I errors, results were followed by a false discovery rate (FDR) threshold of *P*<0.05 to correct for false-positive voxels, and a Monte Carlo Z simulation employing a two-tailed cluster-wise correction threshold of *P*<0.05 to correct for false-positive clusters. Results of both corrections are reported. The individual sub-cortical volume segmentation data were processed in SPSS using an analysis of covariance (ANCOVA) with group as between subject factor and age as a covariate.

In a second structural analysis, we intended to investigate trauma survivors with current light symptom load by excluding the highest scoring participants on the questionnaires until the between-group differences were statistically non-significant.

## Results

Ten of the HC group were recruited as part of another study and only completed the structural scans, leaving 15 HC and 24 TE for the full questionnaire analysis. The remaining participants differed significantly in age (*t*(37)=3.52, *P*=0.001), thus age was included as a covariate. There were no significant differences in BAI, BDI, IES-total or the hyperarousal and intrusion subscales of the IES-R (*F*(1,38)<2.45, *P*>0.126, *η*_*p*_^*2*^<0.066). However, the TE group, compared to HC group, had significantly higher scores on IES-avoidance (*F*(1,38)=4.25, *P*=0.047, *η*_*p*_^*2*^=0.108), but it did not survive Bonferroni correction (*N*=6, *P*<0.006) (Table 1).

**Table 1:** Descriptive statistics for participants completing the questionnaires

	TE		HC	
		
Measures	Mean	SD	Mean	SD
Age	48.38	11.11	33.93	14.40*
Number of females	10		7	
Total	24		15	
BAI	5.20	7.86	3.13	3.72
BDI	4.48	4.93	3.73	3.51
IES-R Sum	13.17	13.04	4.00	8.15
Intrusion	0.82	0.79	0.15	0.34
Avoidance	0.42	0.56	0.12	0.40*
Hyperarousal	0.53	0.66	0.31	0.45

SD, standard deviation; N, number; BDI, Becks Depression Inventory; BAI, Beck's Anxiety Inventory; IES-R, Impact of Event Scale 0—Revised; TE, Trauma-exposed group; HC, Healthy Control group.

* *P*<0.05 (independent sample *t*-test, two-tailed).

### Cortical thickness

Because there were no significant differences between the two groups in the psychometric tests, after corrections, no participants were excluded from the second structural analysis. Thus, only the results from the whole sample are reported.

Cortical thickness data from both hemispheres showed 16 significant clusters at the *P*<0.001 level uncorrected; bilateral superior frontal gyrus and fusiform cortex; left superior parietal gyrus, posterior cingulate cortex, inferior frontal gyrus, and lingual gyrus; and right superior temporal gyrus, inferior temporal gyrus, inferior parietal gyrus, rostral middle frontal gyrus, and pericalcarine cortex (Table 2).

**Table 2:** Cortical thickness between trauma-exposed and controls, uncorrected

Hemisphere	LOG (p^−10^)	Size (mm^2^)	TalX^a^	TalY^a^	zTalZ^a^	Peak region
Left	−3.8	31.79	−20.7	9.6	49.1	Superior frontal g
	−3.48	14.73	−26.6	−52.9	42.3	Superior parietal g
	−3.37	15.3	−9.8	−30.9	40.1	Posterior cingulate c
	−3.09	6.96	−40.4	34	7.8	Pars triangularis
	−3.08	5.88	−13.3	−88.6	−8.9	Lingual g
	3.07	5.54	−38.1	−9	−31.6	Fusiform g
Right	5.29	440.02	40.1	−8.9	−29.3	Fusiform g
	4.73	318.85	44.3	10.7	−29.9	Superior temporal g
	−4.1	37.18	33.3	−51.7	37.5	Inferior parietal g
	−3.95	29.17	8.1	14.5	62.6	Superior frontal g
	−3.82	52.34	19.5	34.8	42	Superior frontal g
	3.71	133.17	53.1	−17.5	−25.9	Inferior temporal g
	−3.58	44.77	31	35.4	22.9	Rostral middle frontal g
	3.36	14.7	9.4	−70.9	6.4	Pericalcarine c
	−3.35	16.54	7.1	20	53	Superior frontal g
	−3.2	18	42.2	−74.6	13.2	Inferior parietal g

LOG(p^−10^), cluster-wise *P-*value where >3 indicates *P*<0.001 and >4 indicates *P<*0.0001. Positive values equal significant greater cortical thickness for TE vs. HC.

a Cluster peaks reported in Thalaraic coordinates.

TE, trauma-exposed group; HC, healthy control group; g, gyrus; c, cortex.

**Fig. 1:**
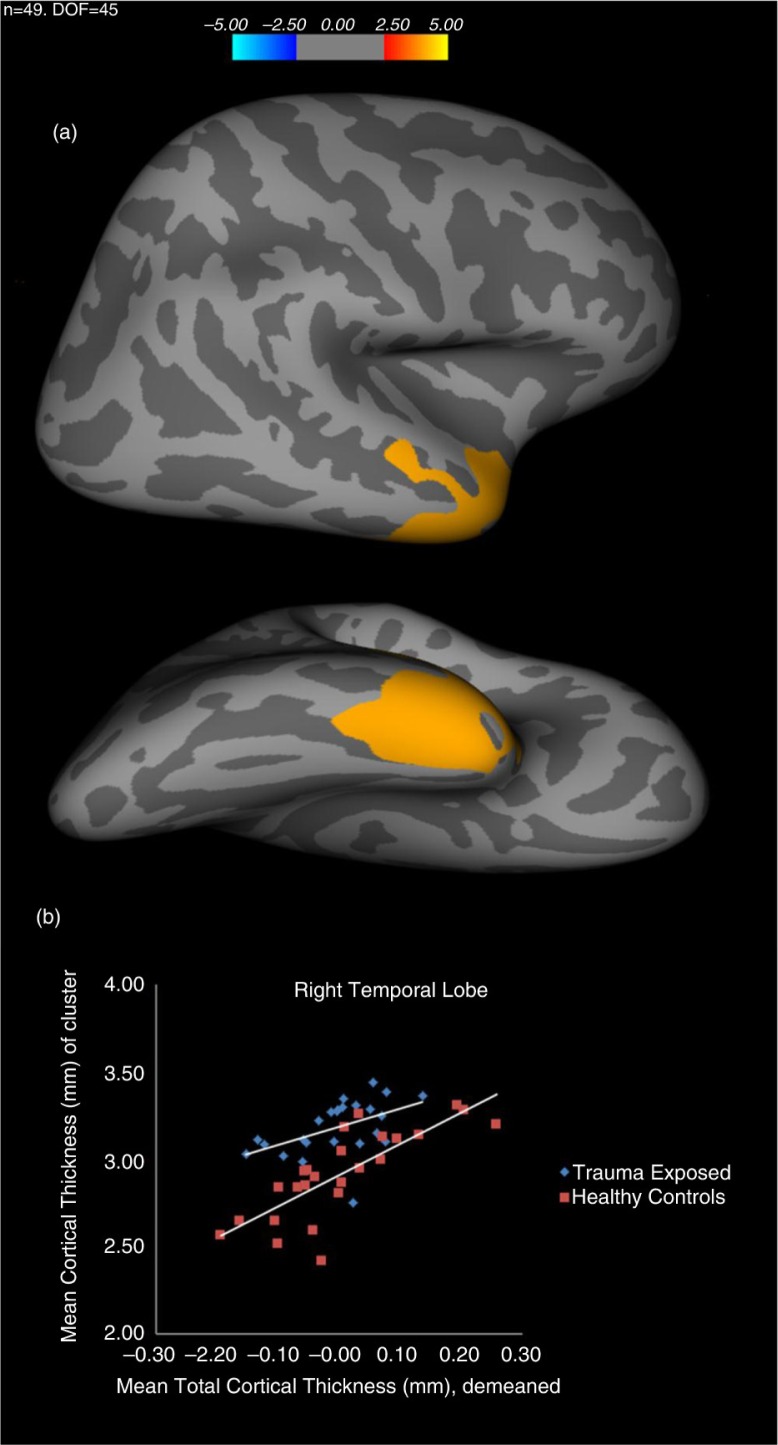
(a) Representation of cortical map depicting greater cortical thickness for the trauma-exposed (TE) group compared to the healthy control (HC) group in superior, middle, and inferior temporal gyrus, and anterior fusiform gyrus. LOG(p^−10^), cluster-wise *P*-value where >4 indicates *P*<0.0001. Top; lateral view. Bottom; ventral view. (b) Scatterplot depicting the mean cluster cortical thickness of cluster (mm) over mean total cortical thickness.

Correcting for an FDR of *P*<0.05 resulted in five clusters: right fusiform gyrus, inferior and superior temporal gyrus, inferior parietal gyrus, and superior frontal gyrus. A Monte Carlo Null-Z simulation with a cluster-wise correction threshold of *P*<0.05 (two tailed) resulted in one cluster (see Fig. 1) centered in the right temporal cortex, including superior, middle, and inferior temporal gyri, as well as anterior fusiform gyrus, indicating an average greater cortical thickness for the TE group (3.18 mm, SD=0.157) relative to the HC group (2.91 mm, SD=0.252). See Table 3 for corrected results.

**Table 3:** Cortical thickness between trauma-exposed and controls, after corrections

Hemisphere	LOG (p^−10^)	Size (mm^2^)	TalX^a^	TalY^a^	TalZ^a^	Peak region
Right^b^	5.29	99.51	40.1	−8.9	−29.3	Fusiform g
	5.08	43.08	49.2	−26.4	−25.5	Inferior temporal g
	4.73	118.23	44.3	10.7	−29.9	Superior temporal g
	−4.1	6.75	33.3	−51.7	37.5	Inferior parietal g
	−3.95	2	8.1	14.5	62.6	Superior frontal g
Right^c^	5.296	3241.24	49.2	−26.4	−25.5	Inferior temporal g

LOG(p^−10^), cluster-wise *P-*value where >3 indicates *P<*0.001 and >4 indicates *P*<0.0001. Positive values equal significant greater cortical thickness for TE vs. HC.

a Cluster peak reported in Thalaraic coordinates

b corrected with a False Discovery Rate threshold of *P*<0.05

c corrected with a Monte Carlo Z-score threshold of *P*<0.05.

TE, trauma-exposed group; HC, healthy control group; g, gyrus; c, cortex.

Subcortical volume data revealed a significant volume difference in the anterior corpus callosum (*F*(1,49)=7.37, *P*=0.009, *η*_*p*_^*2*^=0.138); however, it did not survive a Bonferroni correction of multiple comparisons (*N*=30, *P*<0.0017, *F*>~11).

## Discussion

In this study, we explored morphological differences in trauma survivors of the 2004 Indian Ocean Tsunami. We performed structural MRI scans of 24 TE participants at 7–8 years after the traumatic event, and 25 healthy controls.

After corrections, a cluster encompassing right anterior temporal regions (inferior, middle, and superior temporal gyrus) and anterior fusiform gyrus survived, indicating greater cortical thickness for the TE group when compared to the healthy controls. The TE group also showed greater volume of the anterior corpus callosum and significantly higher scores in the avoidance subscale of the IES-R compared to the healthy controls. While the former has been implicated in PTSD (Kitayama et al., 2007), neither measure was significant after applying a Bonferroni correction of multiple comparisons.

The right temporal lobe has been implicated in several cognitive functions, including explicit long-term semantic memory and abstract concepts (Shimotake et al., 2015), auditory perception (Zatorre, Belin, & Penhune, 2002), emotion perception (Leitman et al., 2010), emotional modality of memory and comprehension (Binder & Desai, 2011), and social cognition (Elzinga & Bremner, 2002; Jou, Minshew, Keshavan, Vitale, & Hardan, 2010), and a recent review of the function of the right anterior temporal lobe implicated it in semantic processing with a bias toward social, emotional, and person-relevant processing (Wong & Gallate, 2012).

In the scope of this study, the emotional semantic memory connection is the most interesting as PTSD is partially modeled as a memory disorder (Brewin, 2011; Elzinga & Bremner, 2002). Several studies implicate semantic processing in PTSD (Pineles, Shipherd, Welch, & Yovel, 2007; McNally et al., 1990; Weber, 2008). One study in particular observed that TE, PTSD-negative participants showed less emotion-induced memory trade-off for emotional items in a memory task, compared to both PTSD-positive and non-TE participants, indicating an emotional semantic encoding resilience factor (Mickley Steinmetz et al., 2012). Memory processes were also implicated in an fMRI study by Geuze Vermetten, Ruf, De Kloet, and Westenberg (2008). They investigated memory formation and retrieval, and observed functional alterations in right lateral temporal regions, among others, that according to the researchers could indicate qualitatively different memory processing in PTSD as compared to TE participants without PTSD. In addition, a twin study by Gilbertson et al. (2006) observed that monozygotic twins of combat veterans with PTSD did not differ on measures of executive function, verbal memory, and verbal learning; however, they did differ compared to monozygotic twins of combat veterans who did not develop PTSD, indicating that lower cognitive abilities including verbal memory, could be a risk-resilience factor. Given the above literature, it is possible that the greater cortical thickness in right anterior lateral temporal regions observed in the present study is associated with greater resilience to trauma due to qualitatively or quantitatively different semantic memory processing. However, while the right temporal lobe is not a common finding in trauma exposure studies (Chen et al., 2012; Eckart et al., 2011; Karl et al., 2006; Kühn & Gallinat, 2013; Patel, Spreng, Shin, & Girard, 2012; Rauch et al., 2006; Shin & Handwerger, 2009), there are some studies implicating the region, although in mostly an opposite direction than the present results.

Woodward, Schaer, Kaloupek, Cediel, and Eliez (2009) observed reduced cortical thickness in the bilateral superior temporal lobes for combat veterans with PTSD compared to those without. A similar study by Geuze, Westenberg, et al. (2008) found increased volume of the bilateral superior middle temporal gyrus. Other studies observed negative correlation between right temporal regions (volume) and flashbacks and re-experience (Kroes, Whalley, Rugg, & Brewin, 2011); and reduced cortical thickness in the right superior temporal gyrus, for motor vehicle accident survivors with PTSD vs. healthy controls (Bing et al., 2013). These findings appear to be consistent with the present results seeing as the observed difference between PTSD negative and control participants would predict lower gray matter thickness for PTSD-positive vs. PTSD-negative participants. Regarding functional imaging, one study by Engdahl et al. (2010) found increased spontaneous resting state functional synchrony in the right temporal lobe. Engdahl and colleagues concluded that this finding reflected earlier studies in cortical electrical stimulation causing “flash-backs” not unlike those observed in PTSD (Penfield, 1958). While the right temporal lobe is not commonly implicated in PTSD, the study by Engdahl might offer an alternative interpretation of the present results.

The present results indicated no significant difference between the groups in terms of symptom load; however, we did not control for past symptom load. Thus, it is possible that the traumatic event might have caused a long-term increase in cortical thickness associated by altered semantic emotional memory processing. In addition, our sample was recruited from a population with overall reported prevalence of PTSD as high as 36.5%, 30 months after the event (Hussain et al., 2011), making it overall very unlikely that none of our participants were at one point diagnosed with PTSD and are now in remission. However, given that the present results are in an opposite direction than results in the PTSD literature (Bing et al., 2013; Geuze, Westenberg, et al., 2008; Kroes et al., 2011; Woodward et al., 2009), it is unlikely that the results are driven by PTSD remitters. In addition, one seminal prospective study found no cortical alterations due to combat stress in combat deployed soldiers without PTSD, as compared to non-combat deployed soldiers (Van Wingen et al., 2011).

Several factors could influence our results. First, the TE group and the healthy controls did not differ significantly on the clinical scales (BDI, BAI, IES-R) except for the IES-R avoidance subscale; an effect that disappears after Bonferroni correction of the six comparisons that were done. This lack of difference in clinical scores, despite a trend toward higher scores for the TE group, could be due to low power caused by non-responders in the healthy control group, thus a larger sample could alleviate this issue, as well as the subgroup differences in age. Second, we assumed that the groups did not differ on past history of trauma exposure, but it was not directly examined. This is a possible confound and could have been measured with the traumatic events questionnaire (Kubany et al., 2000). In addition, measures such as CAPS or PCL-S, could have been used to investigate trauma symptoms more explicitly, however, both measures correlated strongly with IES, BAI, and BDI scores (Adkins, Weathers, McDevitt-Murphy, & Daniels, 2008; Creamer, Bell, & Failla, 2003; Lee et al., 1999). As such, given that the groups did not differ on the BDI, BAI, or IES-R Sum, it is debatable if the two groups differed at all in terms of posttraumatic stress. In fact, only two TE participants scored higher than the IES-R case cutoff of 33 (Creamer et al., 2003). Furthermore, the lack of differences in BDI, BAI, or IES-R indicates that the subjects did not differ in any other comorbid disorders that could confound the observed results. In addition, there was no significant difference in alcohol consumption between the two groups over the last month, as measured by self-rapport. However, we did not control explicitly for past or current psychiatric disorders, or overall health, which might confound results. PTSD remission or past symptom load was also not measured, making it problematic to rule out alternative explanations. A larger sample might have warranted a regression analysis of symptom load against cortical thickness to elucidate the research question further. Third, in terms of generalizability, our sample population was exposed to a single discrete event and quickly repatriated to a high-income European country with an established welfare system, and did not experience secondary disaster stressors, such as destroyed communities or economic loss. A high level of social support and post-trauma care might be an issue in terms of generalizability of our results. In addition, there might be systematic differences between individuals who vacationed in the area, and those who did not, thus education level, intelligence, and socio-economic status should have been measured and controlled for.

Taken together, we observed greater cortical thickness in right anterior temporal regions for participants who experienced a traumatic event 7–8 years prior. This can be indicative that cortical thickness of the right anterior temporal lobe might be a risk-resilience factor, based on quantitative or qualitative differences in processing of emotional semantic stimuli. In addition, our findings might indicate that it is problematic for PTSD studies to only employ a TE or healthy control group, as that might amplify or mask effects. However, due to methodological issues related to sampling and unmeasured possible confounds, replications of the present results are necessary. Furthermore, future research should ideally employ monozygotic twin samples, a pre–post trauma research design, or at least include both PTSD negative and healthy controls as comparison groups.

## Authors' contributions

ASN: main analysis, interpretation, and drafted the manuscript. EH: analysis and revised manuscript draft. NK: acquired data and revised manuscript draft. TH: conceived and designed the study, and revised manuscript draft. EdH: acquired funding, conceived and designed the study, and revised manuscript draft. LL: acquired funding, conceived and designed the study, and revised manuscript draft. TE: acquired funding, conceived and designed the study, and revised manuscript draft.

## Funding statement

This work was funded by South-Eastern Norway Regional Health Authority.
